# Administration of AG490 decreases the senescence of umbilical cord-mesenchymal stem cells and promotes the cytotherapeutic effect in liver fibrosis

**DOI:** 10.1038/s41420-023-01546-3

**Published:** 2023-07-28

**Authors:** Chenhao Jiang, Huaxin Chen, Yinqian Kang, Xinyi He, Jianyang Huang, Tongyu Lu, Xin Sui, Haitian Chen, Jiaqi Xiao, Jiebin Zhang, Hanwen Zhang, Jun Zheng, Yang Yang, Jia Yao, Jianye Cai, Yingcai Zhang

**Affiliations:** 1 Department of Hepatic Surgery and Liver Transplantation Centre, The Third Affiliated Hospital, Sun Yat-sen University, Guangzhou, China; 2 Guangdong Key Laboratory of Liver Disease Research, Guangdong Engineering Laboratory for Transplantation, Guangzhou, China; 3 Biotherapy Centre, The Third Affiliated Hospital, Sun Yat-sen University, Guangzhou, China; 4 Department of Anesthesiology, Sun Yat-Sen University Cancer Center, State Key Laboratory of Oncology in South China, Collaborative Innovation Center for Cancer Medicine, Guangzhou, China; 5 Zhongshan School of Medicine, Sun Yat-sen University, Guangzhou, China; 6 Surgical ICU, The Third Affiliated Hospital of Sun Yat-sen University, Guangzhou, China; 7 Department of Hematology and Medical Oncology, Emory University School of Medicine, Atlanta, GA 30309 USA

**Keywords:** Liver fibrosis, Ageing

## Abstract

The therapeutic potential of umbilical cord-mesenchymal stem cell (UC-MSC) transplantation in liver fibrosis has been highlighted. However, the fate of transplanted MSCs in the fibrotic microenvironment remains unclear. In this study, we aim to uncover the fate of transplanted MSCs and develop targeting strategies that could enhance the therapeutic efficacy of MSC therapy in liver fibrosis. We used human UC-MSCs as the study object. For in vitro experiments, we stimulated UC-MSCs with several fibrotic-related factors (Liver fibrotic Factors, LF), including TGFβ, TNFα and IFNγ for downstream investigations. We co-cultured LF-treated UC-MSCs with hepatic stellate cell line LX-2 to assess the anti-fibrotic effect. We showed that upon LF stimulation, UC-MSCs exhibited reduced anti-fibrotic activity and underwent rapid senescence. Pathway analysis showed that JAK/STAT3 signaling was highly activated upon LF stimulation, which significantly elevated senescence-associated secretory phenotype (SASP) and senescence in UC-MSCs and could be reversed by a specific JAK inhibitor AG490. Moreover, using both carbon tetrachloride (CCl_4_) and 3,5-diethoxycarbonyl-1,4-dihydrocollidine (DDC)-induce fibrosis models, we demonstrated that AG490 pretreatment promoted UC-MSCs survival within the fibrotic liver microenvironment and exhibited enhance therapeutic efficacy. Overall, we showed that targeting MSC senescence in vivo through AG490 pretreatment could enhance the anti-fibrotic activities of UC-MSCs.

## Background

Liver fibrosis is a global disease with high mobility and mortality and is characterized by excessive pro-inflammatory factors and extracellular matrix deposition [1]. Nowadays medical interventions for liver fibrosis focus on the management of risk factors and liver transplantation in patients with the end-stage disease [2, 3]. However, the lack of effective therapies and the limited availability of liver transplantation donors make chronic liver diseases untreatable for most patients.

Recently, transplantation of mesenchymal stem cells (MSCs) has been envisioned as a promising therapy in end-stage liver disease. Evidence shows that MSCs exhibit the capacity of modulating immune response, releasing antioxidants and differentiating into hepatocytes-like in liver disease [4–6]. However, few studies have explored the alteration fate of MSCs under disease-associated microenvironments. Emerging evidence has highlighted the effect of the microenvironment on MSCs and thus influenced the therapeutic efficacy in specific diseases [7, 8]. During liver fibrosis, being chronically exposed to the pro-inflammatory microenvironment and oxidative stress, senescent cells appear to accumulate during liver fibrosis [9–11]. Furthermore, senescent cells have detrimental impacts on neighboring cells through secreting pro-inflammatory cytokines and growth factors, which is known as senescence-associated secretory phenotype (SASP) and contributes to altering the tissue microenvironment and favors disease progress [12].

The Janus kinase (JAK)-signaling transducers and activators of transcription (STAT) signaling pathway was activated in numerous developmental and homeostatic processes [13] and has been reported to be involved in cellular senescence [14]. In our study, we demonstrated that exposing umbilical cord-MSCs (UC-MSCs) to liver fibrosis-related factors, including Transforming Growth Factor beta (TGFβ), Tumor Necrosis Factor alpha (TNFα) and Interferon gamma (IFNγ), which could partially mimic the liver fibrotic microenvironment, resulted in cellular senescence and thus promoted hepatic stellate cells (HSCs) activation. JAK-specific inhibitor AG490 could ameliorate the senescence of UC-MSCs under liver fibrotic microenvironment and promote the therapeutic effect during liver fibrosis.

## Result

### Liver fibrotic factors stimulation weakened the anti-fibrotic capacity of UC-MSCs

**Fig. 1:**
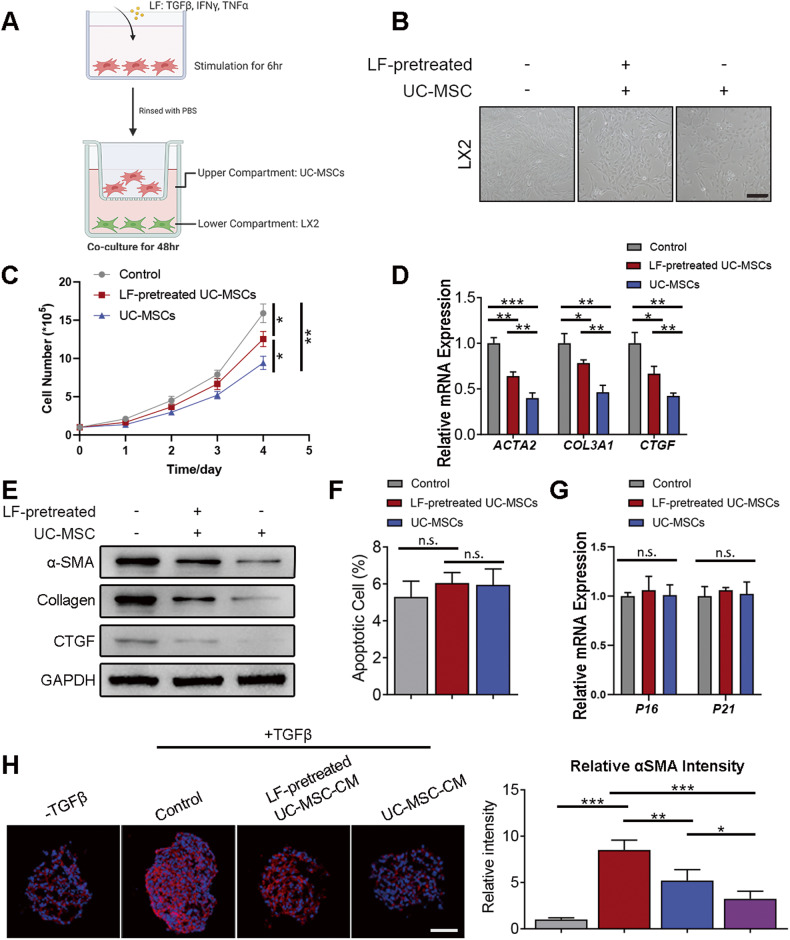
Liver fibrotic factors stimulation weakened the anti-fibrotic capacity of UC-MSCs. **A** Schematic representation of the co-culture system setup. Briefly, UC-MSCs were treated with LF (TGFβ, TNFα and IFNγ) for 6 h before coculturing with hepatic stellate cell line LX2 for 48 h. **B** Bright field imaging of LX2 cells under control condition or co-culturing with differently treated UC-MSCs. Scale bar: 200 μm. **C** viabrate of LX2 with different treatments. **D** Gene expressions of activated HSCs marker assessed by q-PCR in LX2 cells with different treatments. **E** Western blotting analysis of activated HSCs marker expressions in LX2 cells with different treatments. **F** Cell apoptosis ratio of LX2 with different treatments. **G** Gene expressions of senescence marker expressions in LX2 with different treatments. **H** Representative fluorescent images of αSMA (red) in LX2 3D spheroids treated with indicated UC-MSC-CM. Quantifications show the relative αSMA fluorescence intensity. Data are represented as mean ± SEM. *P < 0.05, **P < 0.01, ***P < 0.001.

Previous studies have shown that MSCs exhibit anti-fibrotic effects through paracrine regulation [5, 15, 16]. However, other evidence shown that MSCs produced cues for differentiation of myofibroblast, prevented HSCs from apoptosis, and inhibit the degradation of ECM, which aggravated liver fibrosis. Thus, the fate of MSCs in liver fibrotic microenvironment remains unknown. Firstly, we isolated MSCs from fresh human umbilical cords and cultured as a population of fibroblast-like cells. UC-MSCs were strongly positive for CD73, CD105, and CD90, whereas negative for CD11b, CD45, HLA-DR, CD19, and CD34. In addition, UC-MSCs were successfully induced to differentiate into osteoblasts and adipocytes (Fig. S1). To mimic the liver inflammatory microenvironment of liver fibrosis in vitro, at least partially, UC-MSCs were cultivated with LF factors, including TNFα, IFNγ and TGFβ for 6 h as previously reported [17]. Then, UC-MSCs (with or without LF-pretreatment) were co-cultured with human hepatic stellate cell line LX2 for 48 h (Fig. 1A). Morphologically, while co-culturing with UC-MSCs transformed LX2 into a quiescent state from a more myofibroblast-like phenotype, LF-pretreated UC-MSCs failed to induce LX2 quiescence (Fig. 1B). Moreover, we found that LF-pretreated UC-MSCs was incapable of repressing LX2 proliferation, compared to UC-MSCs group (Fig. 1C). In addition, upregulated expression levels of several fibrotic markers, COL3A1, ACTA2, CTGF, of LX2 were detected in LF-pretreated MSCs co-culture group compared with UC-MSCs group (Fig. 1D, E). Moreover, we showed that LF-pretreated UC-MSCs did not alter apoptosis or senescence level in LX2 (Fig. 1F, G). To further verify our findings, we established a 3D spheroid using LX2, which were stimulated with UC-MSCs-derived condition medium (UC-MSC-CM). Results showed that while normal UC-MSC-CM could alleviate LX2 activation, as revealed by αSMA staining, LF-pretreated UC-MSC-CM was insufficient to produce a similar result (Fig. 1H).

As MSC mainly exerts therapeutic effect through paracrine, we investigated whether the reduced anti-fibrotic activity in LF-pretreated UC-MSCs was caused by an altered secretome. We showed that LF-pretreated UC-MSC-CM was insufficient to inhibit LX2 proliferation as UC-MSC-CM (Supplementary Fig. S2A). Moreover, while LX2 apoptosis and senescence were not altered, the expression levels of HSC activation markers were significantly elevated in LF-pretreated UC-MSC-CM group, compared to UC-MSC-CM group (Supplementary Fig. S2B–D).

In conclusion, we demonstrated that the pretreatment of LF factors weaken the ability of UC-MSCs in transforming LX2 from activated state to quiescent state in LX2 co-culture model.

### Liver fibrosis factors stimulation promoted the senescence of UC-MSCs

**Fig. 2:**
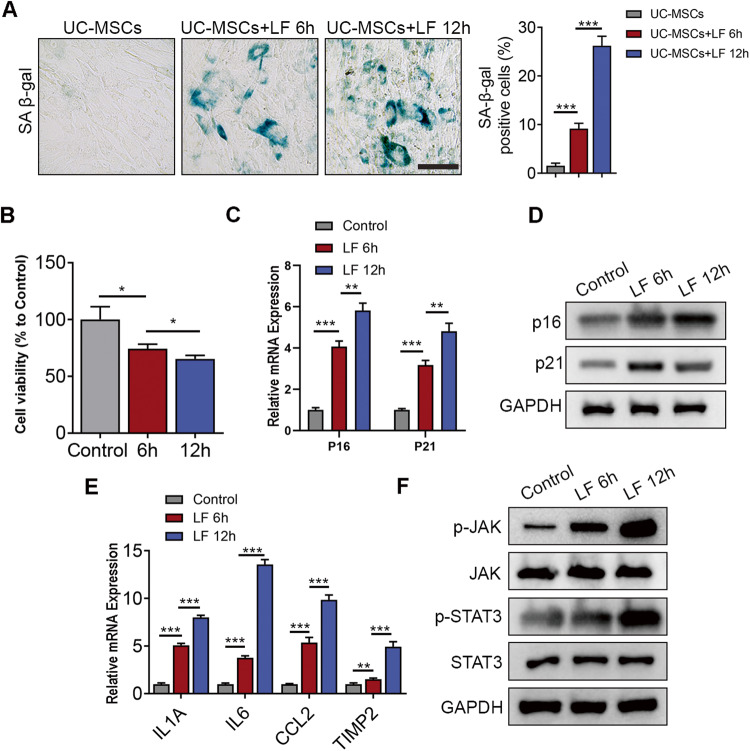
Liver fibrosis factors stimulation promoted the senescence of UC-MSCs. **A** Left panel: Cytochemical evaluation of the senescence marker senescence-associated β-galactosidase activity in UC-MSCs or UC-MSCs + LF for 6 h or UC-MSCs +LF for 12 h. Scale bar: 50 μm. Right panel: Quantification of SA-β-gal-positive cells in the left panel. **B** Quantification of cell viability detected by CCK8 kit. **C** QPCR analysis of several genes related to senescent markers in control and LF-treated UC-MSCs. **D** Immunoblotting analysis of p16 and p21 expression in control and LF-treated UC-MSCs. **E** Gene expressions of SASP marker expressions, including IL1A, IL6, CCL2, and TIMP2, in UC-MSCs with different treatments. **F** Immunoblotting analysis of JAK/STAT signaling pathway activation level in control and LF-treated UC-MSCs. Data are represented as mean ± SEM. *P < 0.05, **P < 0.01, ***P < 0.001.

Next, we sought to investigate the phenotypic alteration of UC-MSCs under LF factors stimulation. Previous studies had demonstrated that pro-fibrotic cytokine TGFβ could induce the senescence of MSCs [18, 19]. Thus, cellular senescence was assessed by quantification of positive SA-β-gal staining [20] and the cellular proliferation was determined through CCK8 (Fig. 2A, B). We observed that LF factors exposure resulted in decreased UC-MSCs proliferation and increased cellular senescence. In addition, increased expression levels of senescence markers p16 and p21 (Fig. 2C, D), as well as SASP markers, including IL6, CCL2, IL1A, TIMP2 were observed in LF-treated group, compared with the control group (Fig. 2E), suggesting that LF factors exposure led to UC-MSC senescence. To verify whether UC-MSC senescence could lead to reduced therapeutic effect, we showed that blocking IL6 signaling, a classical SASP marker using anti-IL6 monoclonal antibody, was sufficient to restore the inhibitory effect of LF-pretreated UC-MSC-CM (Supplementary Fig. S3A, B).

Previous studies have reported that JAK/STAT3 signaling was activated in the senescence of MSCs under fibrotic microenvironment [13, 21]. Indeed, we observed an elevation of JAK/STAT3 signaling after LF stimulation (Fig. 2F). Collectively, these data suggest that the appearance of UC-MSCs senescence was related to the exposure of LF stimulation and associated with the activation of JAK/STAT3 signaling.

### AG490 ameliorated UC-MSCs senescence in liver fibrosis factors stimulation

**Fig. 3:**
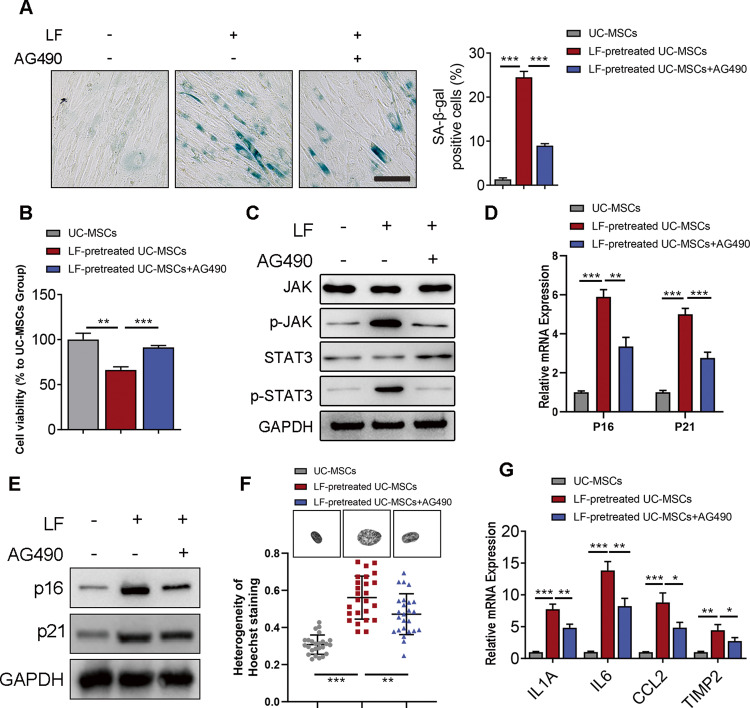
AG490 ameliorated UC-MSCs senescence in liver fibrosis factors stimulation. **A** Left panel: Cytochemical evaluation of the senescence marker senescence-associated β-galactosidase activity in UC-MSCs treated with LF and AG490 or not. Scale bar: 50 μm. Right panel: Quantification of SA-β-gal-positive cells in the left panel. **B** Quantification of cellular proliferation detected by CCK8 kit. **C** Immunoblotting analysis of JAK/STAT signaling pathway activation level in control and senescent UC-MSCs (with or without treatment of LF and AG490). **D** QPCR analysis of p16 and p21 in UC-MSCs treated with LF and AG490 or not. **E** Immunoblotting analysis of p16 and p21 expression in UC-MSCs treated with LF and AG490 or not. **F** Representative images and quantifications of chromatin structure of MSCs shown by Hoechst 33342 staining of the nucleus. Scale bar, 5 μm. **G** Gene expressions of SASP marker expressions, including IL1A, IL6, CCL2, and TIMP2, in UC-MSCs treated with LF and AG490 or not. Data are represented as mean ± SEM. *P < 0.05, **P < 0.01, ***P < 0.001.

In order to determine the specific role of JAK/STAT3 signaling in cellular senescence, we utilized a JAK/STAT3 signaling inhibitor AG490. Administration of AG490 in UC-MSCs under pro-inflammatory microenvironment resulted in a decreased level of positive SA-β-gal staining (Fig. 3A) and a restoration of cellular proliferation of LF-pretreated UC-MSCs group (Fig. 3B). Moreover, we observed an inhibition of JAK/STAT3 signaling pathway in senescent UC-MSCs upon administration of AG490 (Fig. 3C). Furthermore, AG490 reduced the mRNA and protein expression levels of senescence, p16 and p21 (Fig. 3D, E). Besides, we showed that AG490 administration reversed the level of heterogenous chromosome staining in LF-treated UC-MSCs, which is a pivotal characteristic in cellular senescence (Fig. 3F). Consistently, expression levels of SASP-related genes were also decreased with AG490 administration in LF-treated UC-MSCs (Fig. 3G). To conclude, these data suggested that AG490 could ameliorate the senescent phenotype of UC-MSCs through inhibition of JAK/STAT3 signaling.

### Administration of AG490 recovered the anti-fibrotic ability of senescent UC-MSCs

**Fig. 4:**
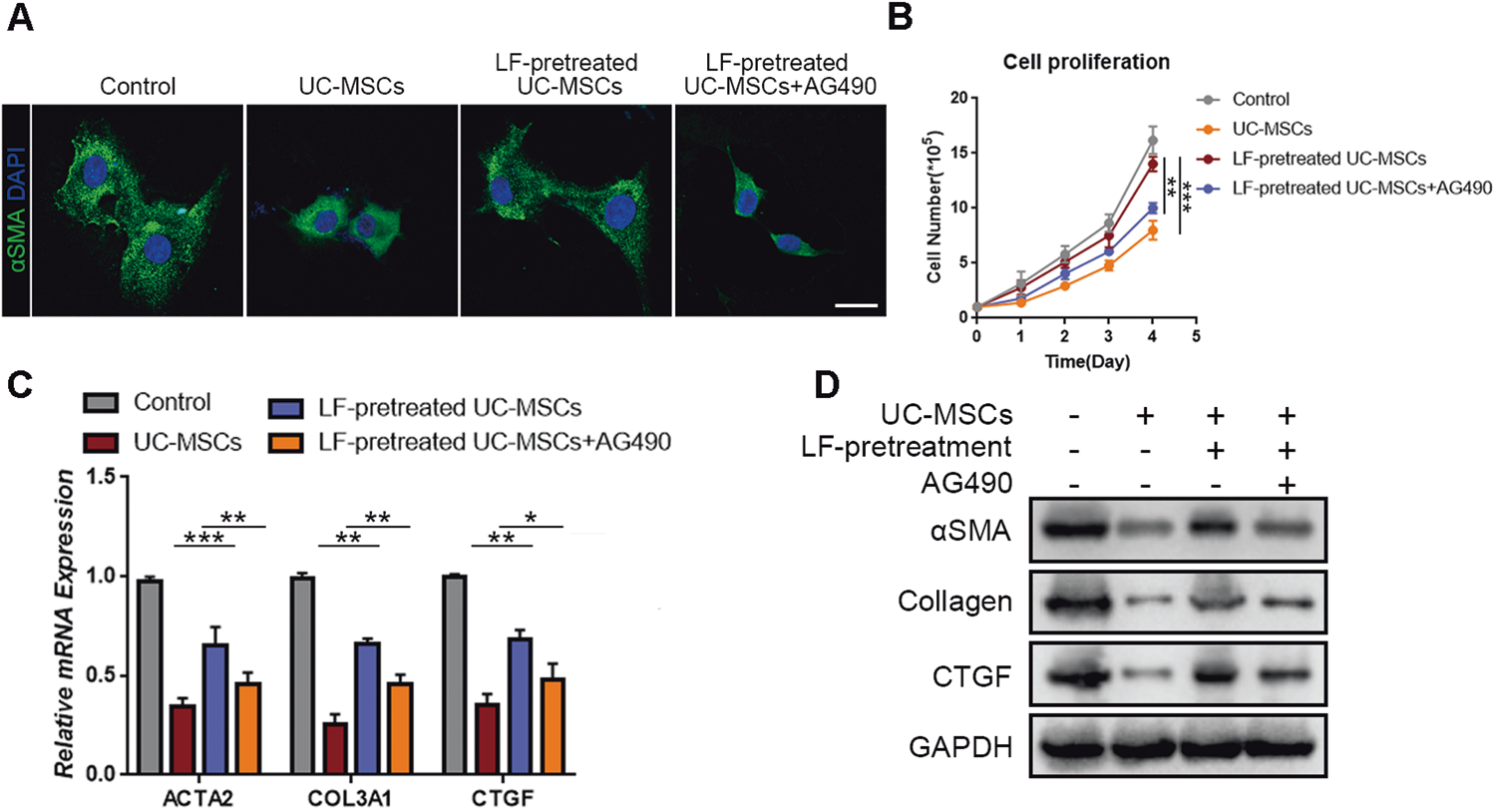
Pretreatment of AG490 recovered the anti-fibrotic ability of senescent UC-MSCs. **A** Representative immunofluorescence images of LX2 cells stained with α-SMA antibody under control condition or in co-culture system with senescent UC-MSCs (with or without pretreatment of AG490). Scale bar: 20 μm. **B** Proliferation rate of LX2 with different treatments. **C** Q-PCR analysis of activated HSC markers in LX2 cells with indicated treatments groups. **D** Immunoblotting analysis of activated HSC markers in LX2 cells with indicated treatments. Data are represented as mean ± SEM. *P < 0.05, **P < 0.01, ***P < 0.001.

We had demonstrated that UC-MSCs underwent senescence after the exposure to LF stimulation. We wonder whether administration of AG490 could recover the anti-fibrotic ability of senescent UC-MSCs. Then, we pretreated LF-treated UC-MSCs with AG490 before co-culturing with LX2 cells. As expected, LX2 cells recovered to a more quiescent phenotype and presented a slower growth rate when co-cultured with AG490-pretreated senescent UC-MSCs compared with senescent UC-MSCs group (Fig. 4A, B). In addition, decreased expression levels of fibrotic markers of LX2 cells were detected in AG490-treated senescent UC-MSCs co-culture group compared with UC-MSCs group (Fig. 4C, D). In conclusion, we demonstrated that AG490 could recover the anti-fibrotic ability of UC-MSCs under LF stimulation, which transformed LX2 cells into a more quiescent state.

### Therapeutic effect of AG490 pretreated UC-MSCs in CCl_4_/DDC induced liver fibrosis

**Fig. 5:**
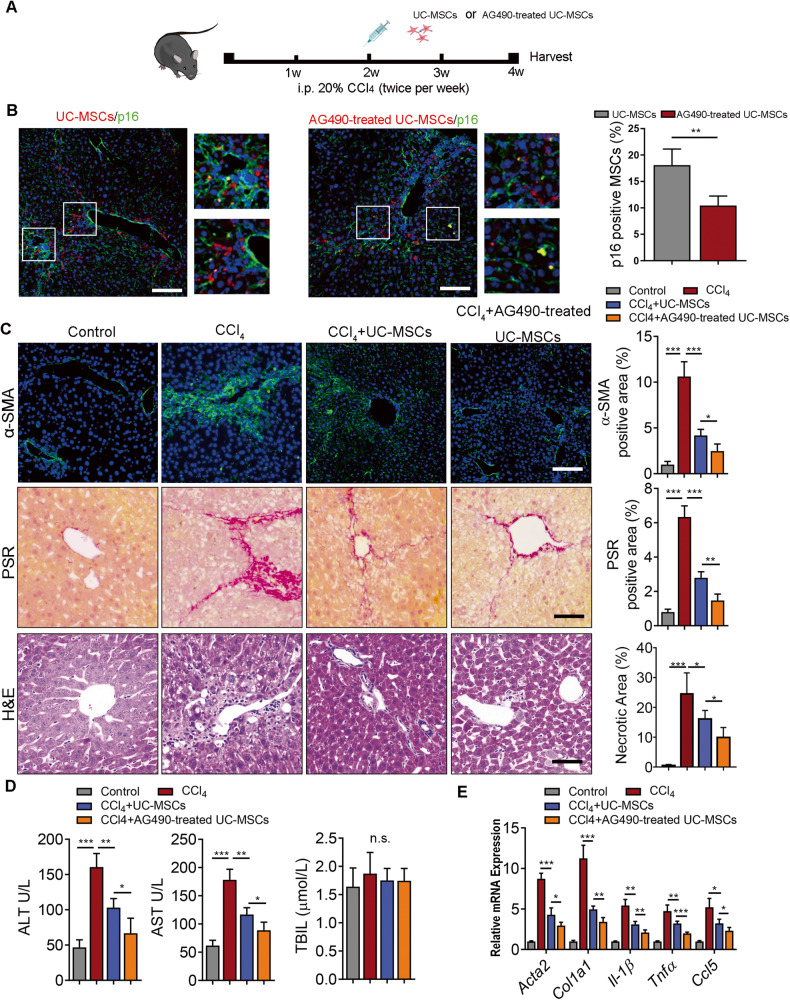
Therapeutic effect of AG490 pretreated UC-MSCs in CCl_4_ induced Liver fibrosis. **A** Schematic representation of the in vivo therapeutic experiment setup. CCl_4_ was administered intraperitoneally twice a week for 2 weeks. Then UC-MSCs with or without AG490 pretreatment were injected via the liver portal vein. Livers were harvested for analysis 2 week after the administration of UC-MSCs or AG490-treated UC-MSCs. **B** Representative immunofluorescence images defining p16 (green) in UC-MSCs transfected with RFP (red) plasmid after the administration of UC-MSCs with or without AG490 pretreatment. Scale bar: 50 μm. **C** Representative images showing α-SMA, PSR and HE staining of liver sections from the indicated groups of mice. Scale bars, 50 µm. **D** Serum ALT, AST, total bilirubin level analysis in mice from the indicated groups. **E** QPCR analysis of fibrotic genes in murine liver tissue following indicated treatments. Each group *n* = 5. Data are represented as mean ± SEM. *P < 0.05, **P < 0.01, ***P < 0.001.

To determine whether AG490-treated UC-MSCs could enhance the cytotherapeutic efficacy in experimental liver fibrosis, we used two well-established liver fibrosis models, the CCl_4_ and DDC models, respectively. For CCl_4_ induced toxic liver fibrosis model, mice were transplanted with 1 × 10^6^ RFP-transfected UC-MSCs after 2 weeks injection of CCl_4_ (Fig. 5A). Using bioluminescence (BLI) imaging, we demonstrated that AG490 pretreatment significantly increased the retention of transplanted UC-MSCs in the fibrotic microenvironment (Supplementary Fig. S4A, B). Transplantation of UC-MSCs or AG490-treated UC-MSCs both showed significantly reduced the liver injury and ameliorated liver fibrosis. In order to confirm the enhanced therapeutic effect of AG490 in UC-MSCs, we co-stained the liver section with p16, the immunohistofluorescence images showed AG490-treatment resulted in reduction of senescent UC-MSCs under local liver fibrotic microenvironment (Fig. 5B). Furthermore, HE, PSR staining and α-SMA staining indicated that levels of inflammatory infiltration, hepatocellular damage and degree of fibrosis were alleviated. However, AG490 pretreatment group showed a better therapeutic effect (Fig. 5C). Indeed, the murine plasma ALT, AST and TBIL levels were also decreased in the AG490-treated UC-MSCs compared with normal UC-MSCs administration group (Fig. 5D). Furthermore, when compared with UC-MSCs group, administration of AG490-pretreated UC-MSCs resulted in a reduction in expression levels of fibrotic genes including *Acta2*, *Col1a1, Il-1β, Tnfα,* and *Ccl5* (Fig. 5E).

**Fig. 6:**
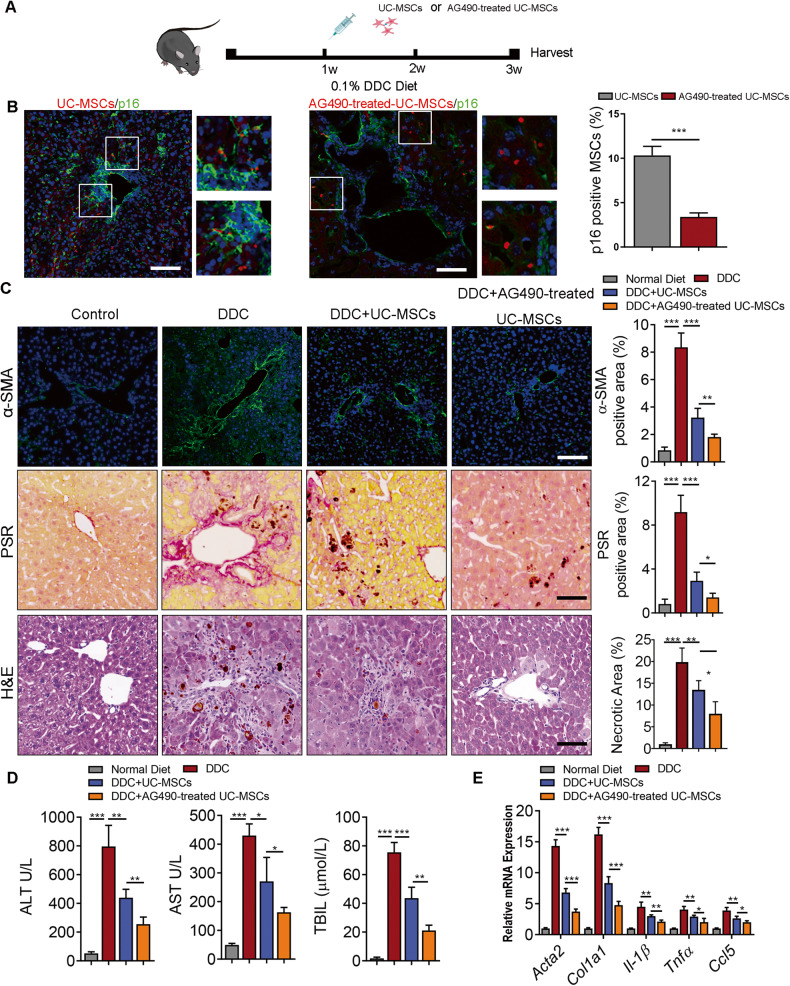
Therapeutic effect of AG490 pretreated UC-MSCs in DDC induced liver fibrosis. **A** Schematic representation of the experiment setup. Mice were fed with 0.1% DDC for 1 weeks. Then UC-MSCs with or without AG490 pretreatment were injected via the liver portal vein. Livers were harvested for analysis 2 week after the administration of UC-MSCs or AG490-treated UC-MSCs. **B** Representative immunofluorescence images defining p16 (green) in UC-MSCs transfected with RFP (red) plasmid after the administration of UC-MSCs with or without AG490 pretreatment. Scale bar: 50 μm. **C** Representative images showing α-SMA, PSR and HE staining of liver sections from the indicated groups of mice after the administration of UC-MSCs with or without AG490 pretreatment. Scale bars, 50 µm. **D** Serum ALT, AST, total bilirubin level analysis in mice from the indicated groups. **E** QPCR analysis of fibrotic genes in murine liver tissue following indicated treatments. Each group *n* = 5. Data are represented as mean ± SEM. *P < 0.05, **P < 0.01, ***P < 0.001.

Similarly, in DDC-induced cholestatic liver fibrosis model, mice were transplanted with 1 × 10^6^ RFP-transfected UC-MSCs after one-week of DDC containing feed (Fig. 6A) . We demonstrated that AG490 pretreatment resulted in a reduced senescent level of UC-MSCs in vivo (Fig. 6B). Besides, AG490-pretreated UC-MSCs displayed an enhanced therapeutic efficacy in DDC-induced liver fibrosis model, as shown by HE, PSR staining and α-SMA staining, compared to normal UC-MSCs (Fig. 6C). Moreover, AG490-pretreated UC-MSCs reduced plasma ALT, AST and TBIL levels (Fig. 6D), as well as expression levels of fibrotic genes including *Acta2*, *Col1a1, Il-1β, Tnfα*, and *Ccl5* (Fig. 6E). In conclusion, these data indicated that AG490 could enhance the anti-fibrotic effect of UC-MSCs in liver fibrosis through reversing the senescence phenotype in UC-MSCs.

## Discussion

**Fig. 7:**
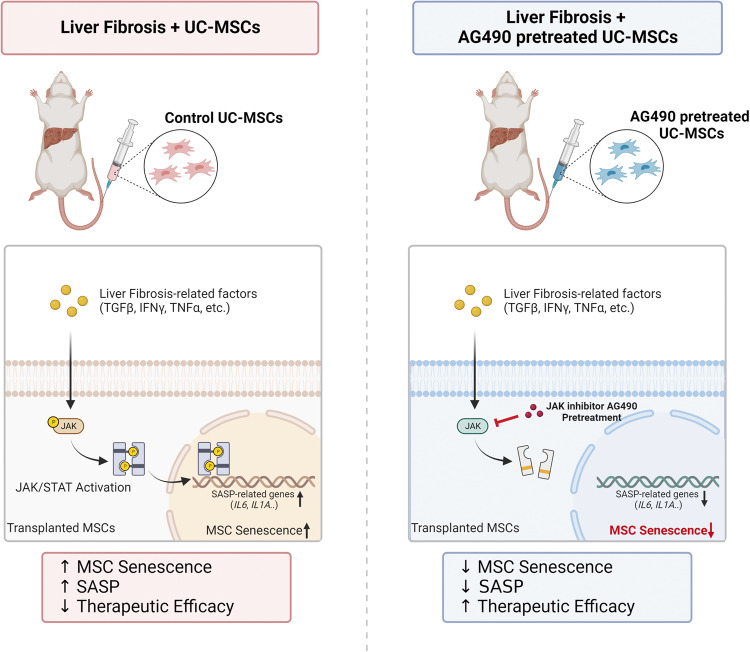
JAK specific inhibitor AG490 pretreatment prevented MSC senescence and promoted therapeutic efficacy in experimental liver fibrosis model.

In this study, we demonstrated that the infused UC-MSCs underwent senescence in the liver fibrosis microenvironment, which significantly hampered the therapeutic effect. Mechanistically, we showed that accumulation of liver fibrosis-related cytokines, including TGFβ, IFNγ, TNFα, etc, could induce UC-MSC senescence and SASP through activation of JAK/STAT3 signaling pathway. Moreover, we demonstrated that targeting JAK/STAT3 through pretreatment of UC-MSCs with a specific JAK inhibitor AG490, could protect UC-MSCs from senescence and further enhance the therapeutic efficacy in liver fibrosis (Fig. 7).

To date, the most effective treatment for terminal liver fibrosis patients is liver transplantation. However, the therapy is limited by scarce donor grafts, immune rejection and the high expenses. There lack effective drugs in reversing the myofibroblasts accumulation and fibrotic microenvironment resulted in the progress to end-stage liver disease. Currently, owing to MSCs capacities of self-renewal, modulating immune response and multiple-lineage differentiation, concerns of MSCs therapy was raised in end-stage liver disease treatment [4, 6].

Emerging evidence demonstrated that transplantation of MSCs could promote hepatocytes proliferation, enhance the activity of matrix metalloproteinases (MMPs), inhibit the activation of HSCs and prompt revascularization of injured liver [22]. Also, MSCs could eliminate the fibrotic microenvironment by modulating injurious immune responses through paracrine pathway [23]. Despite that, there remain numerous concerns in the application of MSCs therapy. For example, different delivery routes and the number of MSCs resulted in various outcome in therapeutic efficacy [19, 24]. Further, low migration of and extension survival of engrafted MSCs resulted from external oxidative stress and the local microenvironment also limited the transplantation efficiency of MSCs [25].

Recently, evidence has highlighted the effect of the microenvironment on MSCs and thus influenced the therapeutic efficacy in specific diseases [7, 8]. In this study, we focus on the fate of MSCs under disease associated microenvironment. There are evidences showing that chronic exposure of diseased associated microenvironment resulted in accumulation of senescent cells [9, 10]. Moreover, through secreting SASP, senescent cells have detrimental impacts on neighboring cells which aggravated disease progress [12]. For example, the MSCs from chronic kidney disease mice showed greater cell senescence upon exposure of oxidative stress [16]. In the study, we observed that the senescent UC-MSCs resulted from fibrotic environment exhibited weaken anti-fibrotic ability through upregulation of SASP. Taken together, better understanding of the interaction of microenvironment around MSCs and the fate of MSCs under microenvironment associated with specific diseases is needed in the future.

In order to improve the MSC transplantation efficacy, various treatments in vitro and in vivo arose. For example, pretreatment of MSCs with a combination of growth factors, cytokine, and chemical compounds prompted hepatocyte-like differentiation of MSCs [26]. Another evidence showed that pretreatment of MSCs with basic fibroblast growth factor (bFGF) markedly enhanced the therapeutic effects on liver fibrosis through increasing the secretion of hepatocyte growth factor (HGF) in MSCs [27]. In the study, we observed that administration of the JAK signaling inhibitor AG490 ameliorated the senescence of UC-MSCs under liver fibrosis microenvironment and enhanced the efficacy of MSCs therapy. In contrary, Arash Pourgholaminejad et al. showed that pro-inflammatory microenvironment enhanced the MSCs capacities of multi-lineage differentiation and immunomodulation [28]. However, based on our results, while MSCs are under inflammatory conditions, the microenvironment didn’t alter the immunogenicity and multi-potency of MSCs but resulted in MSCs senescence. Taken together, our observation may provide new insight in enhancing the efficacy of MSCs under disease associated microenvironment.

To conclude, our findings demonstrate that the liver fibrosis microenvironment resulted in increased MSCs senescence and it proposed that AG490-treated MSCs are better candidate for cell therapy approaches in liver fibrosis. However, in order to established clinical option of MSCs therapy, there still required more clinical studies to increase the safety and reliability of the clinical treatment.

## Methods

### Animal model

#### Fibrosis model

6-week-old male *C57BL/6* mice were purchased from Jiangsu GemPharmatech company, bred under standard specific-pathogen-free conditions. For induction of toxic liver fibrosis, intraperitoneal injection of Carbon Tetrachloride (CCl_4_) (20% CCl_4_ dissolved in corn oil, 5 µl/g of body weight, twice per week) was applied for 2 weeks. For induction of cholestatic liver fibrosis, mice were fed with 0.1% 3,5-diethoxycarbonyl-1,4-dihydrocollidine (DDC) -containing diet for 2 weeks.

#### MSCs treatment model

Mice were divided into 4 subgroups for further MSC treatments: Control group, CCl_4_ or DDC administration group, MSCs treatment group and AG490-treated MSCs treatment group (*n* = 5 for each group). Subsequently, 1.0 × 10^6^ MSCs or AG490-treated MSCs were transplanted into the mice livers via the hepatic portal vein using a 26 G needle. Similar procedure was performed without transplantation in the sham-operated CCl_4_ or DDC group. Liver samples were harvested and analyzed 1 week after the administration of MSCs or AG490-treated MSCs. All procedures were all approved by the Ethical Committee of Sun Yat-sen University and carried out under the guidelines of the Sun Yat-sen University Institutional Animal Care and Use Committee.

### In vivo bioluminescence (BLI) imaging

For in vivo tracking of transplanted UC-MSCs, MSCs of indicated groups were stained with DiR membrane dye for 30 min and washed with PBS. Then DiR-stained UC-MSCs were transplanted into murine liver fibrosis models. At indicated time points, mice were sacrificed and bioluminescence signals were detected with IVIS imaging system (Perkin Elmer). DiR signals were calculated and analyzed by total flux.

### UC-MSCs preparation

Human UC-MSCs were collected upon delivery with informed consent after birth cesarean sections and collected in phosphate-buffered saline at 4 °C. The processing of the umbilical cords and preparation of UC-MSCs were performed at the GMP Stem Cell Laboratory Facility of the biotherapy center in Third affiliated hospital of Sun Yat-sen University according to a well-described protocol [29]. In brief, fresh human umbilical cords were cut into 0.5 cm pieces and floated in Dulbecco’s modified Eagle’s medium (DMEM) containing low glucose, 10% fetal bovine serum (FBS), supplied with 100 U/mL penicillin and streptomycin at 37 °C in a humidified atmosphere with 5% CO2. To remove nonadherent cells, medium was changed every 2 days. After 10 days’ culture, the well-developed colonies of fibroblast-like cells appeared and were trypsinized and transferred into a new flask for further expansion. When UC-MSCs reached 80% confluence, the cells were detached and characterized using fluorescence activated cell sorting (FACS) analysis. For UC-MSC quality control, bacterial, fungal and viral monitoring (including hepatitis B virus [HBV], hepatitis C virus [HCV], human immunodeficiency virus [HIV] and cytomegalovirus) was performed in all umbilical cords. Pathogen free UC-MSCs were cultured and collected at fourth to sixth passages and applied in this experiment procedures.

### In vitro drug administrations

To partially mimic the inflammatory microenvironment of liver fibrosis in vitro, UC-MSCs were stimulated with LF factors, including TGFβ, IFNγ and TNFα. For LF treatment, TGFβ (10 ng/ml, Sigma), IFNγ (50 ng/ml, R&D) and TNFα (20 ng/ml, R&D) were administrated into the culture medium of UC-MSCs. Six hours later, cells were washed, collected and subjected for subsequent experiments. Detailed reagents were listed in Supplementary Table 1.

To explore the role of JAK/STAT3 signaling in MSC senescence as well as anti-fibrotic ability, UC-MSCs were treated with LF factors, followed by JAK/STAT3 signaling inhibitor AG490. For AG490 administration, AG490 was administrated into the culture medium of UC-MSCs at a final concentration of 20 μM. Twenty-four hours later, cells were collected and subjected for subsequent experiments.

### Cell culture and co-culture model

Human hepatic stellate cell line LX-2 was kindly gifted from Professor Shi-Mei Zhuang. Transwells were applied to establish indirect co-culture system. The UC-MSCs were plated on the collagen-coated membrane inserts (24 mm diameter, 0.4 μm pore size; Corning) while the human hepatic stellate cell line LX2 were placed in the lower chamber of a six-well plate at a density of 1.0 × 10^5^ cells/cm^2^ for 48 h. The ratio of UC-MSC and HSC was controlled at 1:1. All cell lines were cultured in DMEM media (Sigma; D5796) with 10% FBS and penicillin/streptomycin (Hyclone) and maintained at 37 °C with humidified atmosphere of 5% CO_2_.

### Heterochromatin structure evaluation

For heterochromatin structure evaluation, cells were stained with Hoechst 33342 (5 μg/ml). Then, cells were analyzed using microscopy. Coefficient of Variation (C.V.) of DNA texture image was used to measure the degree of variation of all pixel value in one nucleus, calculated by the formula (C.V. = Standard deviation / Mean) with ImageJ.

### Senescence-associated β-galactosidase (SA-β-gal) staining

Senescence-associated β-galactosidase kit (Beyotime, C0602) was used for SA-β-gal staining. LX2 cells were incubated with β-galactosidase and X-Gal overnight at 37 °C after fixation in fixative for 15 min. Optical microscope (Leica, DMi8) was applied for following observation of senescent cells.

### Hematoxylin & eosin (H&E) and Picro Sirius Red (PSR) staining

Formalin-fixed paraffin-embedded sections of liver tissues were sectioned into 5 μm-thick sections and stained with H&E and PSR according to standard procedures. Following histologic analyses were performed by the same histopathologist in a blinded manner.

### Immunofluorescence and Immunohistochemistry

The liver tissue sections were prepared following standard steps: fixation with 4% PFA, de-paraffinization, dehydration and antigen retrieval. They were then probed by primary antibody overnight at 4 °C. For immunofluorescence, secondary antibody with fluorescence conjugates were used for detection, followed by 5-min nuclear staining with DAPI. For immunohistochemistry, DAB was used for detection after secondary antibody incubation and Hematoxylin was applied in nuclear staining. LSM780 confocal microscope (Zeiss) and A1R N-SIM (Nikon) were later employed to capture images.

### In vitro UC-MSCs transfection

The plasmid of Red Fluorescent Protein (RFP) transfections performed using the MegaTran 1.0 Transfection Reagent according to the manufacturer’s instructions. The lentiviruses were used to infect UC-MSCs with Polybrene (8 μg/ml) for 6 h. The original medium was replaced with fresh medium 12 h later.

### Cell proliferation assay

Cell proliferation was measured and analyzed by Cell Counting Kit-8 (CCK8, Dojindo, Japan) assay under the manufacturer’s instructions. Briefly, cells were seeded at a density of 1 × 10^4^ cells/mL in 96-well plates. Each well contained 20 µL CCK-8 solution and 200 µL medium. After being cultured for 2 h at 37 °C, the absorbance of each group was detected at 450 nm by an absorbance microplate reader (*n* = 3).

### Western blotting

Proteins were extracted from LX2 cell lysates using RIPA buffer (Millipore) with protease inhibitor cocktail (Roche). BCA Protein Assay Kit (Thermo) were then performed to assess the total protein concentration, 20 μg of which were denatured later and used as samples. The extracted proteins were separated by SDS/PAGE gel and transferred onto PVDF membranes (Millipore, MA) electrophoretically. The target proteins were probed with specific antibodies over night at 4 °C. Supplementary Table 2 consist more information about these antibodies for reference. Chemiluminescent substrate (Millipore) was used for signaling intensity detection after one-hour incubation of membranes with horseradish peroxidase coupled secondary antibody. Bands of GAPDH were used as reference bands for comparison. The original blot are presented in Supplementary Information 2.

### Real-time quantitative-PCR (RT-qPCR)

Total RNA was extracted by TRIzol (Invitrogen, CA, USA) from liver specimens of mice and LX2 cells according to the manufacturer’s instructions. Single-stranded cDNA was then generated from 1 μg of total RNA under the instruction of Revert Aid First Strand cDNA Synthesis Kit (Thermo) and used as templates of qPCR. Based on normalization to the standard housekeeping gene GAPDH, the levels of gene expression were calculated following the ΔΔ CT method. The primers used in qPCR are described in Supplementary Table 3.

### Statistical analysis

All experiments were performed more than three times and results were expressed as mean ± s.e. of the mean (s.e.m.). Comparisons between two groups were performed using the Student’s *t*-test with the help of GraphPad Prism 7 Software. *P* < 0.05 was considered to be significant.

## Supplementary information

Supplemental Material 1

Original Data File

## Data Availability

The datasets used and/or analyzed during the current study are available from the corresponding author on reasonable request.
