# Supplementary material for: Administration of AG490 decreases the senescence of umbilical cord-mesenchymal stem cells and promotes the cytotherapeutic effect in liver fibrosis
Source: Cell Death Discov. 2023 Jul 28;9:273. doi: 10.1038/s41420-023-01546-3 (PMC10382487; doi:10.1038/s41420-023-01546-3)
Supplement: Supplementary file 1 — Supplemental Material 1 [file 41420_2023_1546_MOESM1_ESM.docx]

**Administration of AG490 decreases the senescence of Umbilical cord-****Mesenchymal Stem Cells and promotes the cytotherapeutic effect in Liver fibrosis**

Chenhao Jiang^1^, Huaxin Chen^2^, Yinqian Kang^3^, Xinyi He^4^, Jianyang Huang^4^, Tongyu Lu^1^, Xin Sui^5^, Haitian Chen^1^, Jiaqi Xiao^1^, Jiebin Zhang^1^, Hanwen Zhang^6^, Jun Zheng^1^, Yang Yang^1^, Jia Yao^1*^, Jianye Cai^1*^, Yingcai Zhang^1*^

Supplementary Figure 1-4

Supplementary Table1-3


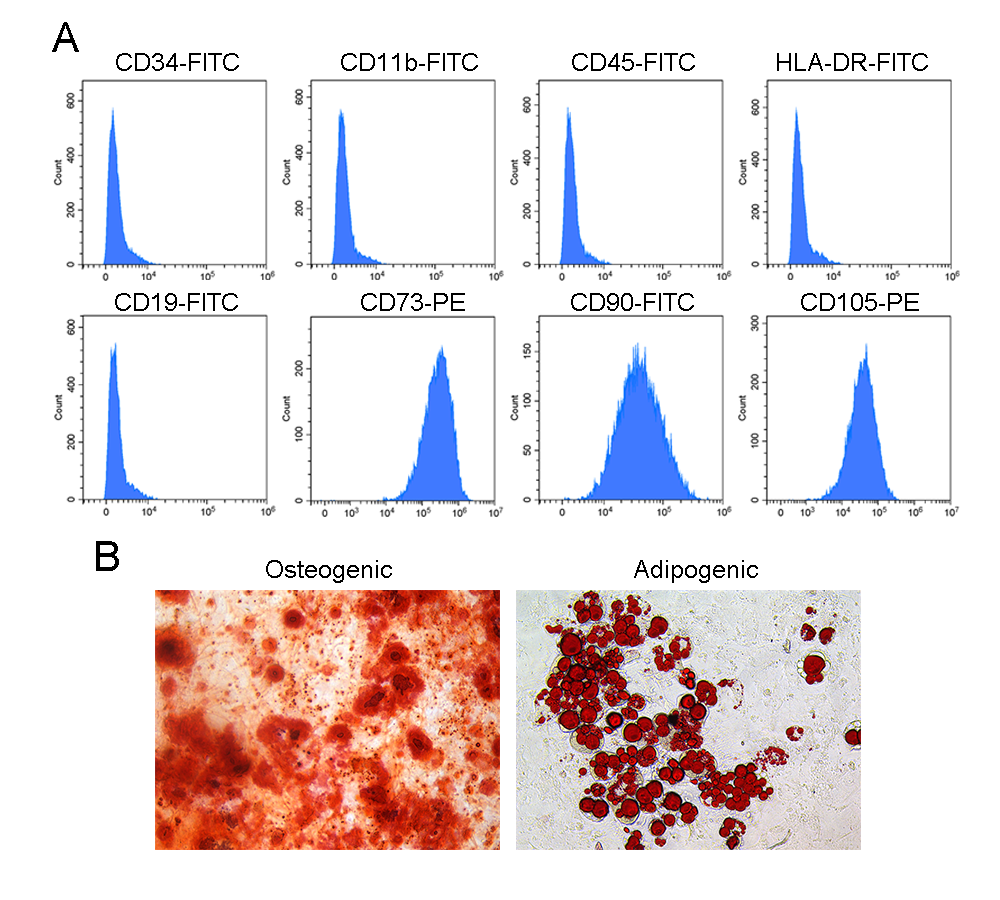


**Supplementary Figure 1. UC-MSCs acquisition and characterization**

(A) Characterization of UC-MSCs by flow cytometry through labeling MSC markers. (B) Multilineage differentiation capacity of UC-MSCs were detect by Alizarin red staining (Osteogenic) and oil red staining (Adipogenic).

**
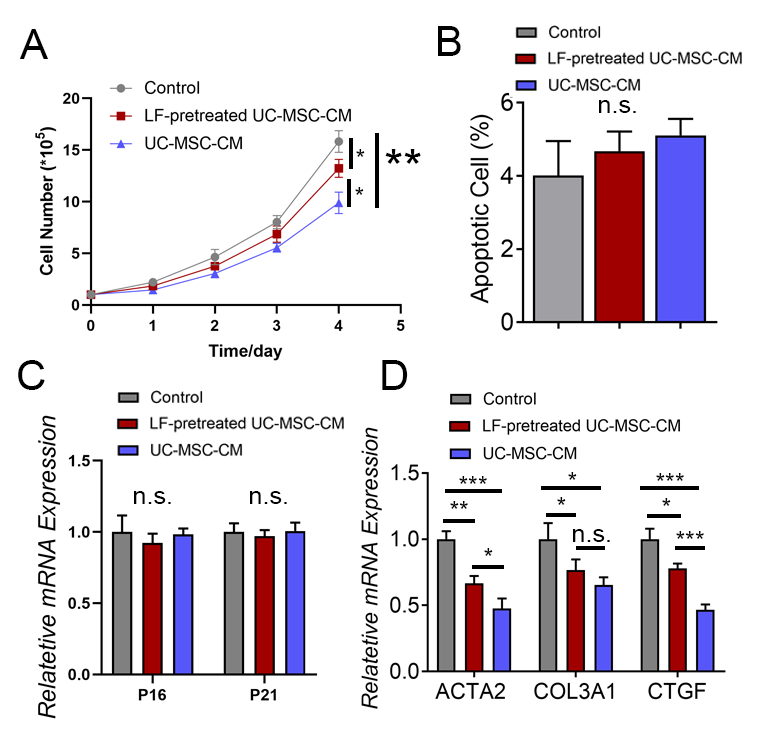
**

**Supplementary Figure 2. LF-pretreated UC-MSC-CM was insufficient to inhibit LX2 activation.**

(A) Proliferation rate of LX2 with different treatments. (B) Cell apoptosis ratio of LX2 with different treatments. (C) Gene expressions of senescence marker expressions in LX2 with different treatments. (D) Gene expressions of activated HSCs marker assessed by q-PCR in LX2 cells with different treatments.


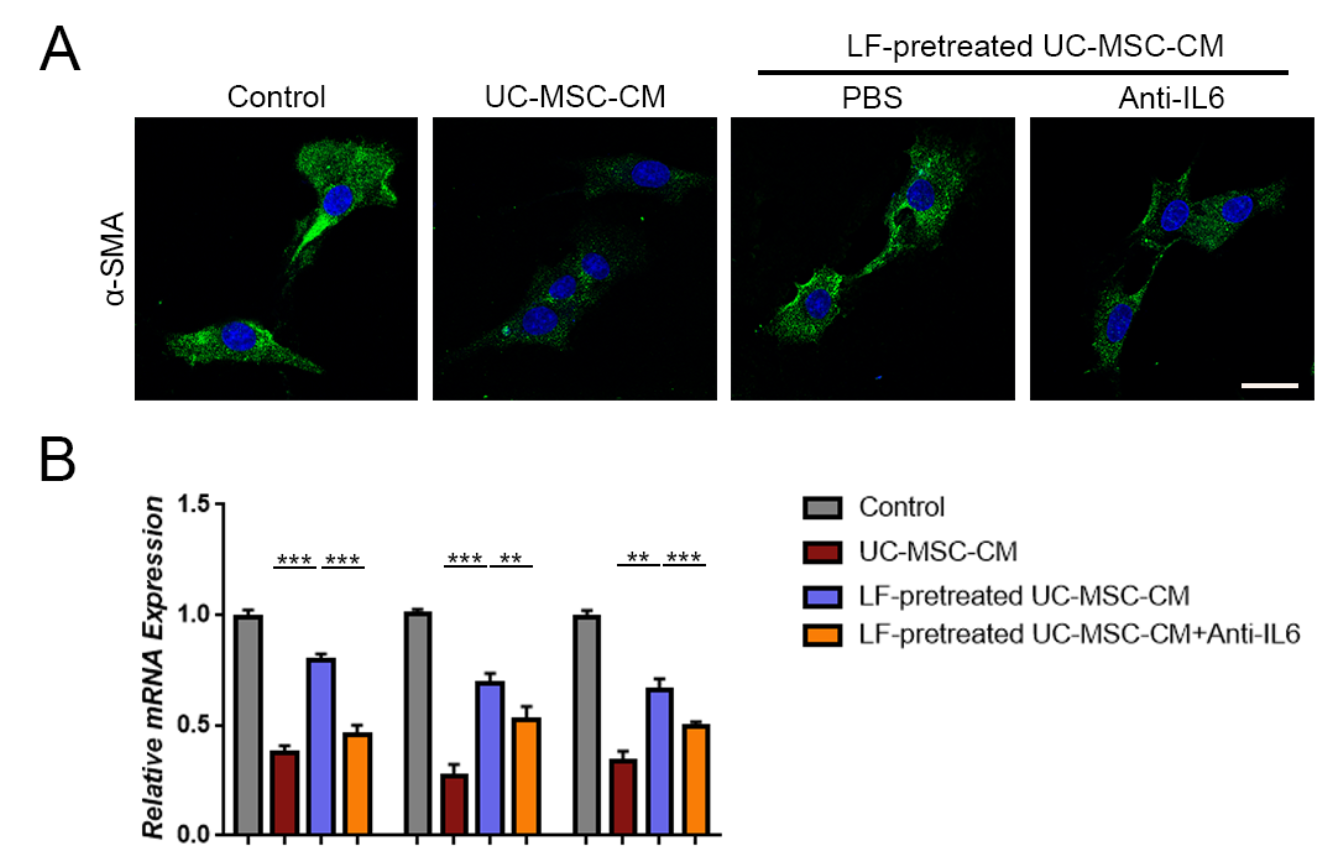


**Supplementary Figure 3. Blockage of IL6 alleviated LX2 activation in LF-pretreated UC-MSC-CM.**

(A) Representative immunofluorescence images of αSMA in LX2 stimulated with LF-pretreated UC-MSC-CM (with or without IL6 mAb administration), Scale Bar=20μm. (B) Gene expressions of activated HSCs marker assessed by q-PCR in LX2 cells with different treatments.


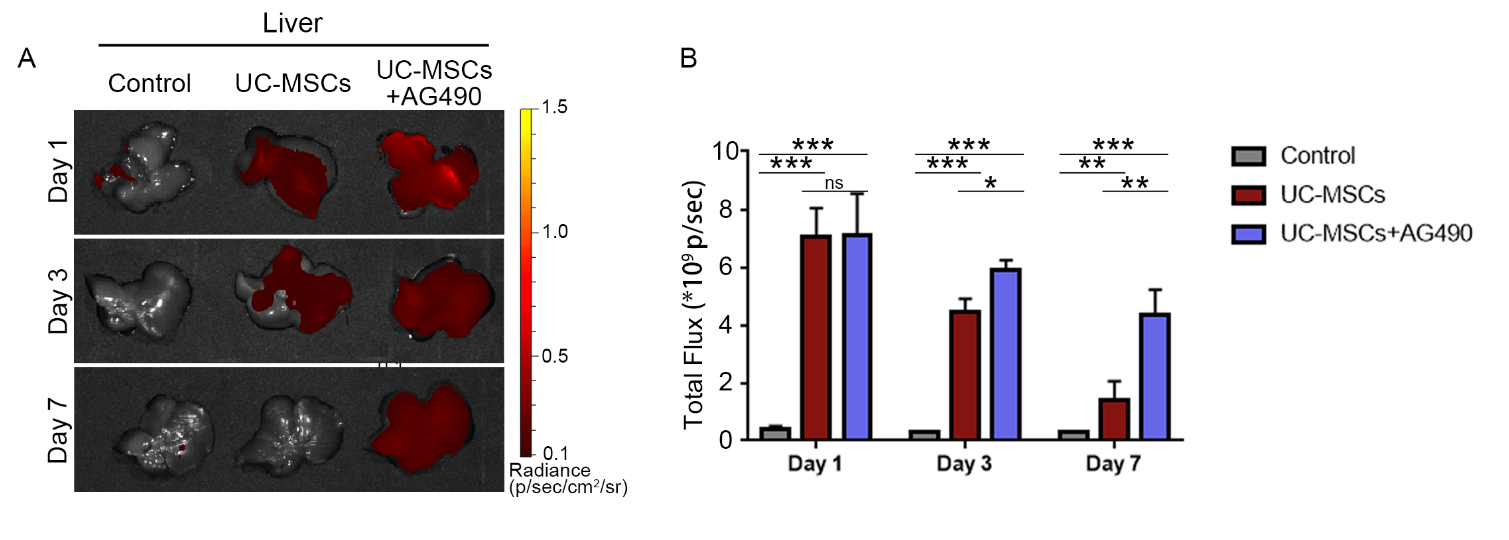


**Supplementary Figure 4. In vivo tracking of transplanted UC-MSCs**

(A) MSCs of indicated groups were stained with DiR dye for 30 min before transplanting into CCl4-induced murine liver fibrosis model. At indicated time points, mice were sacrificed and subjected to *in vivo* bioluminescence (BLI) imaging. (B) Total flux of DiR signal was calculated and analyzed (n=3).

**Supplementary Table 1 Drug and Reagent**

| **Name** | **Supplier** | **Cat no.** |
| --- | --- | --- |
| TGFβ | Peprotech | 93-P1088-10 |
| IFNγ | Peprotech | 96-AF-315-05-20 |
| TNFα | Peprotech | 93-7607-20 |
| PSR staining kit | abcam | ab150681 |
| Siltuximab (anti-IL-6) | Selleck | A2030 |
| DMEM | Hyclone | 12100046 |
| FBS | GIBCO | 10099 |
| MegaTran 1.0 Transfection Reagent | OriGene | TT200002 |

**Supplementary Table 2. Antibody**

| **Name** | **Supplier** | **Cat no.** | **Clone no.** |
| --- | --- | --- | --- |
| Anti-P16 | abcam | 51243 | EP1551Y |
| Anti-P21 | CellSignalTechnology | 2947S | 12D1 |
| Anti-GAPDH | CellSignalTechnology | 2118 | 14C10 |
| Anti-α-SMA | abcam | ab5694 | polyclone |
| Anti-Collagen I | abcam | ab34710 | polyclone |
| Anti-CTGF | abcam | ab6992 | polyclonal |
| Anti-JAK | abcam | ab108596 | monoclonal |
| Anti-p-JAK | Abcam | Ab32101 | monoclonal |
| Anti-STAT3 | Abcam | Ab68153 | monoclonal |
| Anti-p-STAT3 | Abcam | Ab267373 | polyclonal |
| Anti-mouse IgG, HRP-linked Antibody | CellSignalTechnology | 7076 | polyclonal |
| Anti-rabbit IgG, HRP-linked Antibody | CellSignalTechnology | 7074 | polyclonal |
| Anti-mouse IgG2b conjugated to Alexa Fluor 488 | Invitrogen | A-21141 | polyclonal |
| Anti-mouse IgG2a conjugated to Alexa Fluor 594 | Invitrogen | A-21135 | polyclonal |
| Anti-rabbit IgG conjugated to Alexa Fluor 594 | Invitrogen | A-21135 | polyclonal |
| Anti-mouse IgG2b conjugated to Alexa Fluor 488 | Invitrogen | A-21141 | polyclonal |
| Anti-mouse IgG1 conjugated to Alexa Fluor 647 | Invitrogen | A-21240 | polyclonal |

**Supplementary Table 3. Primers for RT-qPCR**

| **Human primer** | **Forward** | **Reverse** |
| --- | --- | --- |
| *P16* | GATCCAGGTGGGTAGAAGGTC | CCCCTGCAAACTTCGTCCT |
| *P21* | TGTCCGTCAGAACCCATGC | AAAGTCGAAGTTCCATCGCTC |
| *COL3A1* | TTGAAGGAGGATGTTCCCATCT | ACAGACACATATTTGGCATGGTT |
| *ACTA2* | AAAAGACAGCTACGTGGGTGA | GCCATGTTCTATCGGGTACTTC |
| *CTGF* | ACCGACTGGAAGACACGTTTG | CCAGGTCAGCTTCGCAAGG |
| *IL6* | ACTCACCTCTTCAGAACGAATTG | CCATCTTTGGAAGGTTCAGGTTG |
| *CCL2* | CAGCCAGATGCAATCAATGCC | TGGAATCCTGAACCCACTTCT |
| *IL1A* | AGATGCCTGAGATACCCAAAACC | CCAAGCACACCCAGTAGTCT |
| *TIMP2* | ACCCTCTGTGACTTCATCGTGC | GGAGATGTAGCACGGGATCATG |
| *GAPDH* | AGGTCGGTGTGAACGGATTTG | TGTAGACCATGTAGTTGAGGTCA |

| **Mouse primer** | **Forward** | **Reverse** |
| --- | --- | --- |
| *18s* | GTAACCCGTTGAACCCCATT | CCATCCAATCGGTAGTAGCG |
| *Col1a1* | TGACTGGAAGAGCGGAGAGT | GTTCGGGCTGATGTACCAGT |
| *Acta2* | CTGACAGAGGCACCACTGAA | CATCTCCAGAGTCCAGCACA |
| *Ctgf* | GGCCTCTTCTGCGATTTCG | GCAGCTTGACCCTTCTCGG |
| *Mmp9* | GCAGAGGCATACTTGTACCG | TGATGTTATGATGGTCCCACTTG |
| *Timp1* | CGAGACCACCTTATACCAGCG | ATGACTGGGGTGTAGGCGTA |
