# Supplementary material for: Administration of AG490 decreases the senescence of umbilical cord-mesenchymal stem cells and promotes the cytotherapeutic effect in liver fibrosis
Source: Cell Death Discov. 2023 Jul 28;9:273. doi: 10.1038/s41420-023-01546-3 (PMC10382487; doi:10.1038/s41420-023-01546-3)
Supplement: Supplementary file 2 — Original Data File [file 41420_2023_1546_MOESM2_ESM.docx]

**Administration of AG490 decreases the senescence of Umbilical cord-****Mesenchymal Stem Cells and promotes the cytotherapeutic effect in Liver fibrosis**

Chenhao Jiang^1^, Huaxin Chen^2^, Yinqian Kang^3^, Xinyi He^4^, Jianyang Huang^4^, Tongyu Lu^1^, Xin Sui^5^, Haitian Chen^1^, Jiaqi Xiao^1^, Jiebin Zhang^1^, Hanwen Zhang^6^, Jun Zheng^1^, Yang Yang^1^, Jia Yao^1*^, Jianye Cai^1*^, Yingcai Zhang^1*^

This file contains original WB blot bands in this research.


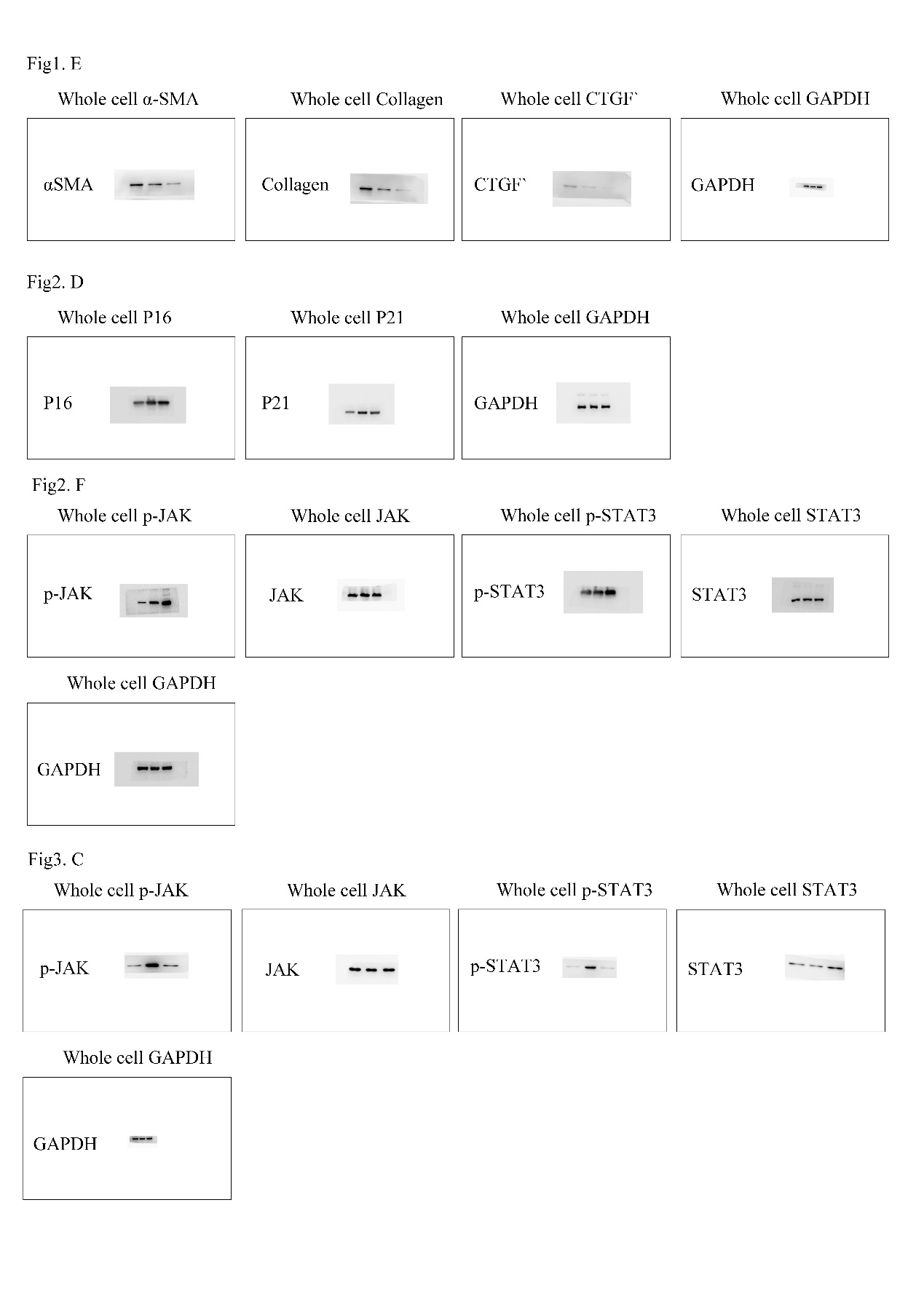


**Original WB blot 1. Related to Figure 1E, Figure 2D and Figure 3C.**


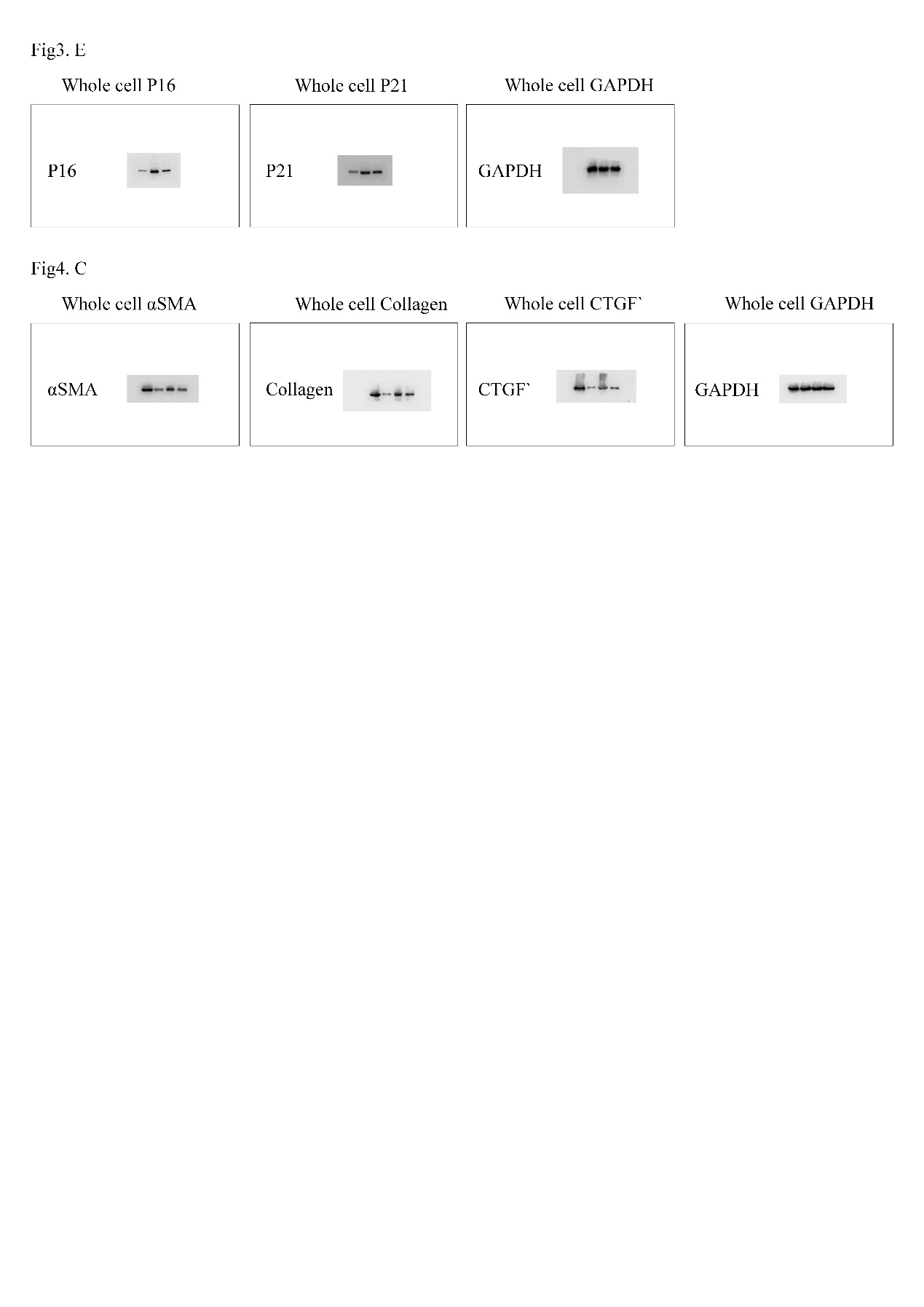


**Original WB blot 2. Related to Figure 3E and Figure 4C.**
